# Combat behaviors predictive of fight outcome in *Gromphadorhina portentosa*

**DOI:** 10.17912/micropub.biology.001927

**Published:** 2026-01-22

**Authors:** Jim J Young, Caleb A Craven, Julianna A Koenig, Merve Addemir, Bin Z He, Olga L Miakotina, Daniel F Eberl

**Affiliations:** 1 Biology, University of Iowa, Iowa City, Iowa, United States

## Abstract

In the winner effect, animals that have previously won an aggressive encounter gain an increased probability of winning subsequent aggressive interactions. This effect has been studied in males of various species, from crickets to humans. However, the effects are under-studied in Madagascar hissing cockroaches, *Gromphadorhina portentosa.* Here, we aimed to determine the influence of the winner effect in unfavorable conflicts. To test this, smaller winner-affected male *G. portentosa* were placed in combat trials against substantially larger males. We tested the limits of the winner effect, and identified behaviors that were predictive to winning a match regardless of treatment group.

**Figure 1. Specific hissing cockroach combat behaviors conducive to winning:**
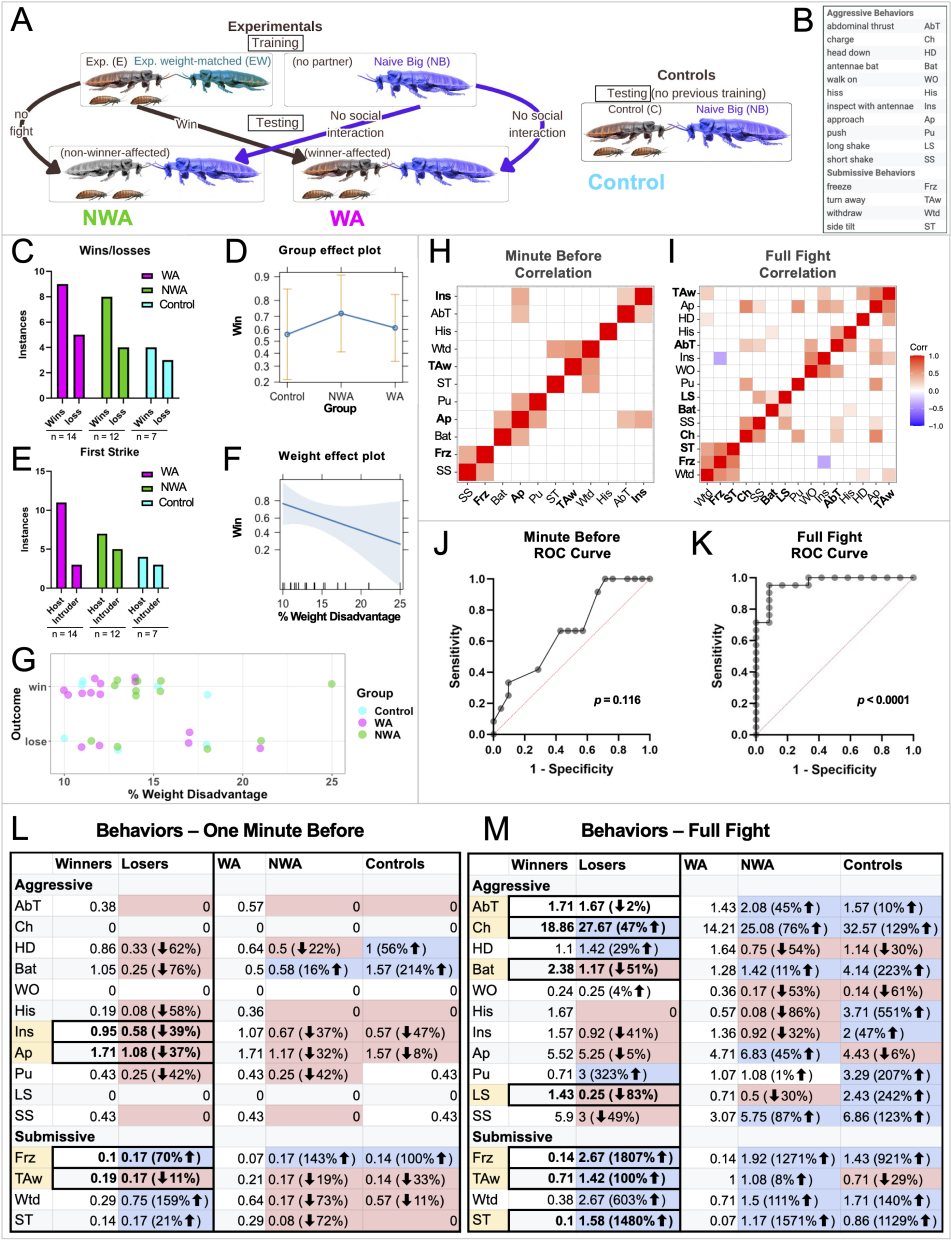
( **A** ) Training and testing flowcharts for Experimental (winner-affected and non-winner-affected) and Control trials. Male cockroaches are depicted according to weight and training outcome. The experimental weight-matched (green) is the training opponent for the experimental male. The males that did not fight in their training session were labeled as non-winner-affected (grey) males in their testing fight. The males that fought and won their training session were labeled as winner-affected males (natural color). Control males (natural color) were not given a training fight. All three categories of males WA, NWA and Controls were placed against a naïve big (purple) cockroach. All experimental and control cockroaches had two female cockroaches below them at reduced scale, signifying the presence of females in their territory during residency establishment and fights. ( **B** ) List of all aggressive and submissive behaviors. ( **C** ) Total number of wins and losses was not significant between winner-affected, non-winner-affected, and control group (Fisher’s exact test, two-tailed, *p* > 0.9999). ( **D** ) The match results are fit to a logistic regression model with group and weight as the explanatory variables. Shown are the predicted probabilities in each group averaged over all weight differences. Error bars represent 95% confidence intervals. ( **E** ) Total number of host and intruder first strikes was not significant between winner-affected, non-winner-affected, and control group (Fisher’s exact test, two-tailed, *p* = 0.454). ( **F** ) Predicted probabilities of winning shown as a function of the weight difference between the experimental animal and the naive big, averaged across the three treatment groups. The light blue shaded area represents a 95% confidence band. ( **G** ) Plot for the binary outcome of win or loss for the three treatment groups by percent weight disadvantage. ( **H, I** ) Spearman’s correlations between pairs of behaviors recorded shown as a heatmap from minute before ( **H** ) and over the course of the full fight ( **I** ). Red indicates a positive correlation and blue indicates a negative correlation between variables. ( **J, K** ) Receiver Operating Characteristic (ROC) curve for the minute before ( **J** ) and full fight ( **K** ) from multiple logistic regression. Win/lose outcome was set as the response variable and the behaviors in either set were the independent variables. Data from all three treatment groups were combined. Representative variables of freeze, approach, turn away, and inspect were selected from correlation matrix clusters for the minute before ( **J** ). Representative variables of side tilt, charge, antennae bat, abdominal thrust, turn away, long shake, and freeze were selected from the full fight correlation matrix clusters ( **K** ). ( **L, M** ) Losers show lower frequencies of aggressive behaviors and higher frequencies of submissive behaviors compared to winners. Aggressive and submissive behaviors during the minute before ( **L** ) and full fight ( **M** ) for each testing fight of winner-affected, non-winner-affected and control subjects. Every aggressive and submissive behavior ( **B** ) was recorded during the minute before and for the entirety of each fight after the first charge occurred. The average frequency of each behavior per individual was calculated. The tables compare the behavioral frequencies of winners to losers. The loser frequencies are colored red if lower than winner frequencies, and blue if they are higher. The table also compared WA to NWA and control behavioral frequencies. The NWA and C frequencies are colored blue if they are higher than WA and red if they are lower. The behaviors highlighted yellow were used in our multiple logistic regressions. The percentage difference between groups for each behavior was calculated by the following: ((Higher Frequency - Lower Frequency)/ Lower Frequency) * 100%. Percentage difference could not be calculated if either frequency was zero. For WA, n = 14, for NWA, n = 12 and for controls, n = 7. Winners n = 21 and losers n = 12.

## Description

The winner effect is observed in the behavior of many species of animals, both vertebrate and invertebrate. The effect is demonstrated when an animal that was previously victorious against a conspecific goes on to display a higher likelihood of winning subsequent fights (Yan et al., 2024). Two main theories for the mechanisms responsible for the winner effect are the self-assessment hypothesis and the social-cue hypothesis (Rutte et al., 2006). According to the self-assessment hypothesis, individuals experiencing the winner effect demonstrate a heightened evaluation of their own fighting ability, gaining a greater familiarity of the energy costs of a conflict (Hsu et al., 2009). The social-cue hypothesis proposes that winner-affected males exude a social signal that discourages rivals from committing to a fight. A study analyzing the winner and loser effect in killifish found that individuals of previous conflicts may display alterations to behavior, morphology, or chemical signaling that is detectable by potential opponents who change their approach to the contest as a result (Hsu et al., 2009). Furthermore, evidence exists that the winner effect in certain species is entirely dependent on biological compounds. This was demonstrated in cricket experiments in which blockers for octopamine receptors effectively nullified the winner effect (Rillich and Stevenson, 2011).

The duration of the winner effect has been found to vary dramatically between species, from as short as 20 minutes in crickets (Rillich and Stevenson, 2011) to as long as 48 hours in mangrove killifish, *Rivulus marmoratus* (Hsu and Wolf, 1999). Winner effects have also been seen to disappear and reappear in a span of 5 weeks in lobster cockroaches (Kou et al., 2019). One species in which the winner effect has been understudied is the Madagascar hissing cockroach ( *Gromphadorhina portentosa)* . Previous studies found hissing cockroaches that won a fight 60 minutes prior to their second fight were more likely to win, demonstrating the presence of the winner effect in this species (Mack, 2022). However, many characteristics of the winner effect have yet to be studied in *G. portentosa,* including its influence on unfavorable fights, hormone involvement, and effect duration. Conflicts between *G. portentosa* males strongly favor the heavier male (Clark and Moore, 1995). In the absence of the winner effect, when one male is distinctly heavier than the other, the lighter male is expected to deem aggression too costly and withdraw without escalation (Parker, 1974). In jumping spiders, *Phidippus clarus* , heavier males are also favored and exhibit the winner effect (Elias et al., 2008). When both size and experience were tested together in *P. clarus* males, weight was found to be 1.3 times more important than experience (Kasumovic et al., 2009). In our study, we hypothesize that males with the winner effect would display more aggression and win unfavorable fights against heavier males at higher rates than non-winner-affected naïve males, and non-winner-affected males that recently were exposed to a size-matched male.

We set up our experiment to maximize aggression displayed by *G. portentosa* to most clearly observe behavioral changes demonstrated under the influence of the winner effect when in unfavorable conflicts. To test them during their most active time of day, we ran our aggression experiment during their dark cycle. Each male was isolated for two weeks to establish residency in his novel territory and fasted one week prior to conflict trials as arthropods fighting over food or territory are known to increase in aggression when fasted (Scharf, 2016). Host males were housed with two females in the winner-affected (WA), non-winner-affected (NWA) experimental, and control (C) groups ( Figure 1A ). Fights were exclusively conducted in the host’s resident enclosures. Territorial status and presence of females were previously shown to heighten inter-male aggression (Guerra and Mason, 2005). Before experimental males were tested against heavier males, they underwent training matches against weight-matched males in order to induce the winner effect. The winners of these training matches were tested against 10-25% heavier males 10 minutes after the conclusion of individual training matches. Experimental males that were introduced to a weight-matched male yet did not engage in a fight were categorized as NWA and progressed to the heavier male combat trial ( Figure 1A ). Observations of winner-affected, non-winner-affected, and control groups were compared with one another to look for differences in their fight outcomes and the behaviors each group displayed before and during the trials that may have contributed to those outcomes ( Figure 1C- M). One of the caveats associated with our design is the self-selection bias outlined by Yan et al. (2024). While that meta analysis did not find a statistically significant difference between self-selection and random selection study results, we agree that future experiments should proceed with a random assignment testing method for better assurance that the winner/loser effects are directly attributable to winning and losing.

Analysis of the raw number of wins and losses between groups found that although males with prior experience (WA and NWA) won noticeably more matches than they lost, these differences between wins and losses were not found to be significant ( Figure 1C, Fisher’s exact test, *P* > 0.9999). Thus, we cannot reject the null hypothesis that the fight outcome is independent of the treatment. Supporting this, the group effect plot shows that regardless of whether a subject is winner-affected, non-winner-affected, or naïve, the probability of winning was negligibly different between groups ( Figure 1D ). These results suggest that the sample size of our experiment does not give us sufficient power to reject our null hypothesis that&nbsp;there is no difference in the probability of winning among groups. ( Figure 1C- G). Conversely, host males tended to strike first, especially in the winner-affected group ( Figure 1E ). This trend corresponds to an explanation in previous literature for the winner effect wherein winner-affected males are more likely to strike first, and males that strike first are more likely to emerge victorious (Mack, 2022). One possibility for why the win rate was non-significant may be that the winner effect was insufficient in overcoming higher weight disadvantages. Results of the binary plot demonstrate only a subtle trend of winner-affected males primarily winning in matches with lower weight disadvantages, between 10 and 15 percent ( Figure 1G ). Furthermore, the weight effect plot portrays a negative correlation between win proportion and weight disadvantage ( Figure 1F ). This ultimately reinforces previous findings of weight being a strong predictor of fight outcome (Kasumovic et al., 2009). Winner-affected males also trended towards fewer charges ( Figure 1M ) while no significant difference in fight duration was seen compared to controls. In contrast, Syrian hamsters (Huhman et al., 2003) and Mediterranean field crickets, *Gryllus bimaculatus* (Rillich and Stevenson, 2011) that had previous contest victories went on to display an increased number of attacks in future contests.

Exhaustion may have contributed to our experimental outcome. Of the total trials run, 8 out of 15 controls and 15 out of 29 experimental males did not fight (no interaction with the intruder). While these trials were not included in our data, their number supports patterns observed in previous literature. Aggression related to fasting in arthropods typically has a bell-shaped curve relationship - after a certain period of time, a decline in aggression is observed (Scharf, 2016). The one-week fast to increase aggression may have been too long, producing opposite effects. The experimental males may have been depleted of energy from their training fight, leaving them less likely to challenge the heavier intruder. Additionally, it is possible that the 10 minute inter-fight intervals between their size-matched fight and their testing fight may have not given them enough time to regain their energy. These two factors may have contributed to the lower number of aggressive actions observed in the WA hosts when compared to control and NWA hosts, who competed against the heavier intruder without previous exhaustion.

We next switched the focus of our study to the identification of specific behaviors that predict winning. Since we didn’t observe a significant winner effect, all three treatment groups were combined for this analysis to achieve a higher statistical power. This resulted in a total of 33 trials. We quantified 11 aggressive behaviors and 4 submissive behaviors in two time periods: one minute before (MB) and full fight (FF) ( Figure 1B ). Therefore, we have a total of 30 behavior traits. To assess the linear dependence between these traits, we performed Principal Component Analysis on the standardized data matrix by centering and scaling the values within each trait variable. Three traits were removed because the corresponding behaviors were not observed during the minute before period, resulting in zeros across all trials (Ch, WO, LS). Of the remaining 27 variables, the top 10 Principal Components (PCs) explained 82.9% of the total variance, suggesting that many traits are highly correlated. To select representative variables for the subsequent regression analysis, we calculated Spearman correlation coefficients for all pairwise comparisons within each of the two time periods and arbitrarily selected variables (bold text, Figure 1H, 1I) from each correlation block. To test if the behaviors before or during the full fight were predictive of the outcome, we performed multiple logistic regression with the variables selected above for each time period (Table 1). The ‘minute before’ behaviors were found to be not or only weakly predictive of the fight outcome (Area under the Receiver Operating Characteristic, or ROC Curve = 0.67, *p* = 0.116, Figure 1J ). Of the four behaviors chosen, subjects with higher frequencies of inspect, approach and turn away had positive correlations to winning their fight while freeze had a notable correlation to losing ( Figure 1L ). The behaviors we included in the minute before period in the multiple logistic regression were not predictive of fight outcome. Thus, under our experimental conditions we failed to find support for the social cue hypothesis.

**Table:** 

**Table 1. Results of multiple logistic regression on selected correlated behaviors for two time periods.** A) Log odds ratio estimates of the selected minute before behaviors to predict fight outcome from the combined data of all three groups (WA, NWA, Control). (Hosmer Lemeshow hypothesis test, *p* = 0.453; Area under ROC curve, *p* = 0.116, n = 33). *In support of Figure 1J . * B) Log odds ratio estimates of the selected full fight behaviors to predict fight outcome from the combined data of all three groups (WA, NWA, Control). (Hosmer Lemeshow hypothesis test, *p* = 0.839; Area under ROC curve, *p* < 0.0001, n = 33). *In support of Figure 1K . *				
**A. Behaviors 1 Minute Before Fight**				
Odds ratios	Variable	Estimate	95% CI (profile likelihood)	
β0	Intercept	0.6359	0.1259 to 2.817	
β1	FrzMB	0.7222	0.06681 to 7.995	
β2	ApMB	1.647	0.7151 to 4.445	
β3	TAwMB	1.343	0.1806 to 13.62	
β4	InsMB	1.518	0.6091 to 5.631	
**B. Behaviors During Full Fight**				
Odds ratios	Variable	Estimate	95% CI (profile likelihood)	
β0	Intercept	93.01	1.165 to 2122521	
β1	ChFF	0.9323	0.8269 to 1.018	
β2	AbFF	1.436	0.7620 to 3.177	
β3	TAwFF	0.06350	0.0004134 to 0.5643	
β4	LSFF	2.541	0.02945 to 443.2	
β5	STFF	2.328	0.01528 to 243.4	
β6	BatFF	7.591	1.330 to 325.0	
β7	FrzFF	0.0196	4.100e-005 to 0.4759	

By contrast, the set of selected ‘full fight’ behaviors were strongly predictive of the outcome, as shown by a statistically significant area under the ROC curve (area = 0.96, *p* < 0.0001, Figure 1K ). Of these behaviors, long shake and antennae bat were observed at higher frequencies in subjects that won their fight while subjects with a higher frequency of charge, turn away, side tilt, and freeze were more likely to lose ( Figure 1M ). The statistically significant results from the full fight ROC curve allow us to better understand what set of behaviors are predictive of fight outcomes against heavier opponents (Table 1). The full fight behaviors had substantially more influence on outcome than minute before behaviors suggesting that social cues in *Gromphadorina* produced before the start of the fight have less influence on the outcome of the fight than the behaviors used during the fight *.*

In summary, we observed a slight trend toward winner affected males overcoming a weight disadvantage of up to 15%. We also discovered that our selected behaviors used before the start of the fight were not predictive of fight outcome, suggesting the social cues we observed in the host males before the fight had less influence on the outcome than the behaviors they used during it. On the other hand, abdominal thrusts, long shakes, antennae bat, charges, side tilts, turning away, and freeze were found to have the greatest predictive power towards fight outcome. Previous studies have determined the presence of the winner effect in *G. portentosa* . Our study identifies specific behaviors that contribute to fight outcome. We found no evidence for pre-fight social cues influencing the outcome of the fight under the conditions used in our study.

## Methods

*Animals*

We used data from 158 adult Madagascar hissing cockroaches, *Gromphadorhina portentosa* , including 33 host males (14 WA, 12 NWA, 7 C), 26 experimental weight-matched (EW) males, 33 large males (NB), and 66 females ( Figure 1A ). These numbers do not include the 15 experimentals and 8 controls that did not fight. Animals were sourced from colonies maintained at 22-24 **°** C and 44-51% relative humidity. We provided shelter (paper towel rolls), food (Harland irradiated laboratory animal diet #503) when the animals were not fasted, and unlimited water.

*Preparation of males*

Individual males were isolated in 7-quart plastic containers including lids with holes for aeration for 2 weeks. Each container had a toilet paper roll for shelter, a water source (small glass vial with cotton plug), and one pellet of rat chow. Each host male was housed with 2 females. One week before trials, food was removed to heighten aggression (Scharf, 2016). Isolation enclosures were housed under 8L:16D light cycle.

Males in the WA, NWA, and Control groups (host males) were selected to be within 5% body weight of each other. Bigger males were selected to be 10-25% heavier than host males.

*Description of behaviors*

Below we describe the 13 behaviors that we assessed in this study. The description includes the name of each behavior, the abbreviation in parentheses (see Figure 1B ) followed by the description and published source.

Aggressive Behaviors:

- Abdominal Thrust (AbT)- lift lower abdomen up in quick motion (Mack, 2022).
- Charge (Ch)- Lower head and rapidly hit other animal with horns (Mack, 2022).
- Head down (HD)- lowering head below carapace while standing still (Mack, 2022).
- Antennae bat (Bat)- animated sword fighting with antennae (Breene III, 2014).
- Walk on (WO)- climb on top of an opposing male (Mack, 2022).
- Hiss (His)- production of distinct hissing sound in the presence of other males (Clark and Moore, 1994).
- Inspect with antenna (Ins)- contact with the antennae (Clark and Moore, 1994).
- Approach (Ap)- directed movement toward another individual (Clark and Moore, 1994).
- Push (Pu)- Lower head and slowly push on opposing male with horns (Mack, 2022).
- Long shake (LS)- winner of social interactions wiggle their abdomen over ground or on an opponent (Barth, 1968).
- Short shake (SS)- vigorously shake abdomen during fight (Mack, 2022).

Submissive Behaviors:

- Freeze (Frz)- lower body against substrate and remain motionless (Clark and Moore, 1994).
- Turn away (TAw)- avert direction of body away from opponent after confrontation (this study).
- Withdraw (Wtd)- moving away from an individual and/or an interaction (Clark and Moore, 1994).
- Side tilt (ST)- lower head, lean body toward opponent (Mack, 2022).

&nbsp;

*Combat Trials*

Host males were kept in their isolation enclosures which served as the arena for combat trials. Experimental trials were initiated 2 hours into the dark cycle under red light. Water and shelter were removed and a barrier was introduced to decrease the area of the arena to 30-40%. Before each trial, the intruder male was hand-held for acclimation for five minutes to reduce transfer stress. The intruder male was then allowed to crawl off the hand into the host's enclosure at least 2 inches away from the host. For the experimental males, there was a 10-minute period between the termination of their training fight against a weight-matched male and the introduction of the heavier male. Experimental males that lost their training match were excluded from the experimental pool. Experimental males that had a training match set up but did not participate in it were labelled as non-winner affected (NWA). These NWA males, like winner-affected males, were subjected to their testing fight after a ten minute interval despite non-engagement in their size-matched trial. For training and test fights, behaviors of both males were video recorded for 10 minutes or until there was a conclusive winner. Winners were identified through clear victory displays (long shake) or an opponent retreating. The host’s behaviors were assessed and tallied for each test fight according to the descriptions in the previous section. In addition, we noted time taken to initiate fight, duration of conflict, and which male initiated conflict.

*Statistical analysis*

Principal Component Analysis was performed in GraphPad Prism v10.5. Data were standardized by centering and scaling within each variable. Three variables, Ch, WO and LS in the minute before period had zeros in all trials, and thus were removed from the PCA analysis. Pairwise Spearman’s correlations were calculated among variables in each of the two time periods. Variables were selected manually by examining the hierarchically clustered correlation heatmap ( Figure 1H, 1I) so that one variable per block of highly correlated traits were picked arbitrarily to represent that group.

Multiple logistic regressions were performed to test whether the selected representative behaviors were predictive of the fight outcome. All three treatment groups (WA, NWA, C) were consolidated into a single data set with dependent variable set as win/loss and independent variables set as behaviors from either minute before or full fight ( Figure 1J, K). Win/loss was set as a categorical variable while all other behaviors were set as continuous variables. “Lose” was set as “negative outcome” in analysis. The model only contained an intercept and all the main effects. No interaction terms were included. Area under the Receiver Operating Characteristic (ROC) Curve and the associated *p* -values as well as odds ratios were reported ( Figure 1J, K, & Table 1).

Logistic regression of wins by weight disadvantage and group was conducted using R. Win/loss against weight by group was then plotted using a ggplot ( Figure 1G ). We fit the models for main effects (group + weight = model 1) and checked goodness of fit (Hosmer-Lemeshow). With the odds ratios and confidence intervals of model 1 (Table 1), we created a group effect plot to depict win probabilities for each group over all weight differences ( Figure 1D ). Similarly, we generated a weight effect plot with the combined data from all three groups to depict the predicted probability of winning as a function of weight difference between the host male and the heavier opponent ( Figure 1F ). A 95% confidence interval was included for all data in both plots.

3x2 tables were made for both host/intruder first strike and wins/losses for all three test groups (WA, NWA, C) and analysed using two-tailed Fisher’s exact tests ( Figure 1C, E).

We conducted a post-hoc power analysis to determine sample sizes that would have been necessary to show a statistically significant difference between WA and Controls in match outcome. We used the Chi-squared sample size calculator at https://homepage.univie.ac.at/robin.ristl/samplesize.php with the observed win probability difference (WA: 9/14≈0.64; Controls: 4/7≈0.57; difference =0.07) as the effect size, and 0.05 as the critical value. This effect size is understandably smaller than seen previously (~0.59) in fights between size matched individuals and comparing winners to losers (Mack 2022). The required sample size to achieve 0.8 power for our results would be 765. A similar analysis for testing the "experience effect", where we combine WA and NWA as the "experienced" group and contrast it with Controls, resulted in an observed winning probability of 17/26≈0.65 and a similar sample size required to achieve 0.8 power. In both cases, our sample sizes are grossly inadequate for such a small effect size.&nbsp;
